# High-resolution energy data from a sustainable industrial production area in Karlsruhe

**DOI:** 10.1038/s41597-026-06955-4

**Published:** 2026-02-28

**Authors:** Jonas Sievers, Simon Bischof, Thomas Blank, Frank Simon

**Affiliations:** Karlsruhe Institute of Technology, Institute for Data Processing and Electronics (IPE), 76344 Eggenstein-Leopoldshafen, Germany

## Abstract

Understanding and optimizing industrial energy systems requires datasets that capture detailed electrical behavior at high temporal resolution over long time periods. Such data are essential for analyzing power quality, identifying operational patterns, and developing data-driven models for forecasting, control, and predictive maintenance. Yet, most existing open datasets lack the temporal granularity, measurement diversity, and machine-level detail needed to reflect the complexity of industrial environments. To address this gap, we present a large-scale, high-resolution dataset of industrial electricity measurements comprising more than 74 billion data points collected at 5-second resolution over up to seven years. The dataset includes 22 industrial machines and one photovoltaic system, with up to 190 measured quantities per device, including three-phase voltages and currents, active, reactive, and apparent power, harmonic spectra, total harmonic distortion, and fundamental waveform characteristics. In addition, the dataset is complemented by external metadata such as weather, electricity prices, and emission factors. This unique combination of long-term coverage, high sampling rate, and rich feature space enables insight into industrial energy dynamics and provides a robust foundation for advancing machine learning, digital twins, and intelligent energy management in industrial environments.

## Background & Summary

The industrial sector accounts for approximately 40% of global electricity consumption in 2024^1^, highlighting its central role in the transition to efficient and low-carbon energy systems. Unlike residential or commercial buildings, industrial facilities exhibit complex and highly variable electricity profiles shaped by heterogeneous machinery, production cycles, and operational constraints^2^. Power-electronic equipment such as drives, converters, and lighting systems further contribute to this complexity by acting as nonlinear loads that introduce voltage distortion and harmonic currents^3^. In such environments, electrical behavior directly influences process stability, equipment lifetime, and efficiency, requiring a detailed understanding of machine-level power characteristics for effective energy management and control.

Building on this need, data-driven methods are increasingly important for industrial energy research and operation. Forecasting and optimization aim to anticipate load dynamics and improve scheduling efficiency^4^, while anomaly detection and predictive maintenance identify deviations that threaten process stability or equipment health^5^. Digital twins integrate synchronized voltage, current, and power quality data to reproduce system behavior under real operating conditions^6^, and flexibility assessment quantifies the temporal and contextual boundaries for demand-side management^7^.

The effectiveness of these approaches depends on measurement data that combine high temporal resolution, extended duration, and detailed power-quality information at the machine level. However, such datasets remain scarce. Most publicly available data originate from residential or commercial domains and lack the granularity, duration, and diversity required to represent industrial processes. To systematically assess these limitations, we review representative open datasets across both residential and industrial contexts, focusing on temporal resolution, measurement period, disaggregation level, and power quality coverage (see Table 1). Here, power quality refers to the deviation of voltage and current from a pure sine wave, as measured by harmonics and total harmonic distortion (THD). Although this work focuses on machine-level industrial measurements, we include residential and commercial datasets to highlight potentially useful data sources for method development and transfer to industrial settings.

**Table 1:** Concept matrix for the literature on energy datasets across residential (R), industrial (I), and tertiary (T) sectors.

Dataset	Domain	Entity	Resolution	Duration	Real Data	Power Quality
Ausgrid^8^	R	300 Buildings	30 minutes	3 years	◉	
REDD^9^	R	5 Buildings, 92 Devices	1 second	119 days	◉	
UK-Dale^10^	R	5 Buildings, 111 Appliances	1 second	6 years	◉	
REFIT^11^	R	20 Buildings, 160 Appliances	8 seconds	2 years	◉	
Low Carbon London^13^	R	5,567 Buildings	30 minutes	3 years	◉	
German Households^14^	R	38 Single-Family Houses	Non-uniform	2,5 years	◉	◉
iFlex^15^	R	4,483 Households	1 hour	1 year	◉	
IDEAL^16^	R	255 Homes in UK	Non-uniform	23 month	◉	
ELMAS^12^	I + T	424 Electrical Load Profiles	1 hour	1 year	◉	
Industrial Park in China^17^	I	4 Facilities	5 minutes	6 years	◉	
EWELD^18^	I	386 Buildings and Machines	15 minutes	6 years	◉	
South Korean Factories^19^	I	10 Factories	1 minute	7 months	◉	
Paper and Food Industry^20^	I	3 Factories	1 hour	2 years		
HIPE^21^	I	11 Machines	5 seconds	3 months	◉	◉
This paper	I	22 Machines + 1 PV	5 seconds	7 years	◉	◉

Residential datasets are typically characterized by predictable consumption patterns and well-defined appliance categories. The Ausgrid dataset offers three years of 30-minute resolution load and rooftop photovoltaic (PV) generation data from 300 Australian households, supporting energy forecasting or PV integration^8^. For device-level modeling, REDD^9^, UK-Dale^10^, and REFIT^11^ provide sub-minute resolution data, including aggregated and appliance-level loads. These datasets enable studies on non-intrusive load monitoring (NILM), load disaggregation, and residential forecasting. ELMAS^12^ includes one year of hourly data from 424 French industrial and tertiary sectors, supporting energy efficiency analyses. The Low Carbon London dataset^13^ complements these sources by providing 30-minute electricity consumption for 5,567 Greater London households from November 2011 to February 2014, including a 2013 dynamic time-of-use trial with roughly 1,100 customers that received day-ahead high, low, and normal price signals to study residential demand response and tariff-driven load shifting. The dataset from Schlemminger *et al*.^14^ offers high-resolution electrical load profiles from 38 German single-family houses. The dataset records apparent, active, and reactive power, as well as voltage, current, and power factor, at temporal resolutions ranging from 10 seconds to 60 minutes over a 2.5-year period. To explore consumer response to market signals, the iFlex dataset^15^ provides one year of hourly electricity consumption from 4,483 Norwegian households participating in a dynamic pricing experiment, supplemented by extensive survey data on socio-demographic variables and willingness to provide flexibility. The IDEAL household energy dataset^16^ provides a comprehensive view of 255 homes in the United Kingdom over 23 months, featuring 1-second electricity data alongside room-level environmental measurements and occupant surveys to facilitate research into energy demand drivers and NILM.

In contrast, publicly available industrial datasets remain scarce. The dataset from an industrial park in China offers six years of five-minute data from four buildings, supporting long-term consumption analysis in clustered industrial sites^17^. EWELD includes 15-minute data over six years from 386 industrial and commercial users, annotated with extreme weather events such as typhoons and heatwaves, enabling load forecasting under adverse conditions^18^. A dataset from 10 manufacturing sites in South Korea provides one-minute electricity consumption data over seven months, focusing on demand response participation and virtual power plant design^19^. Additionally, a synthetic dataset for the Chilean paper and food industry offers two years of hourly profiles from three factories, facilitating energy system modeling^20^.

A prominent publicly available dataset for industrial energy research is the High-resolution Industrial Production Energy (HIPE) dataset^21^, which provides 5-second electrical measurements from 11 machines in a real production environment over a three-month period in 2017. It includes active power and selected power quality indicators and has been widely used for studies on load disaggregation, power quality assessment, and demand-side management. Despite its impact, the dataset’s short duration and limited scope restrict its suitability for long-term analysis, reliability assessment, and advanced data-driven modeling.

**Fig. 1:**
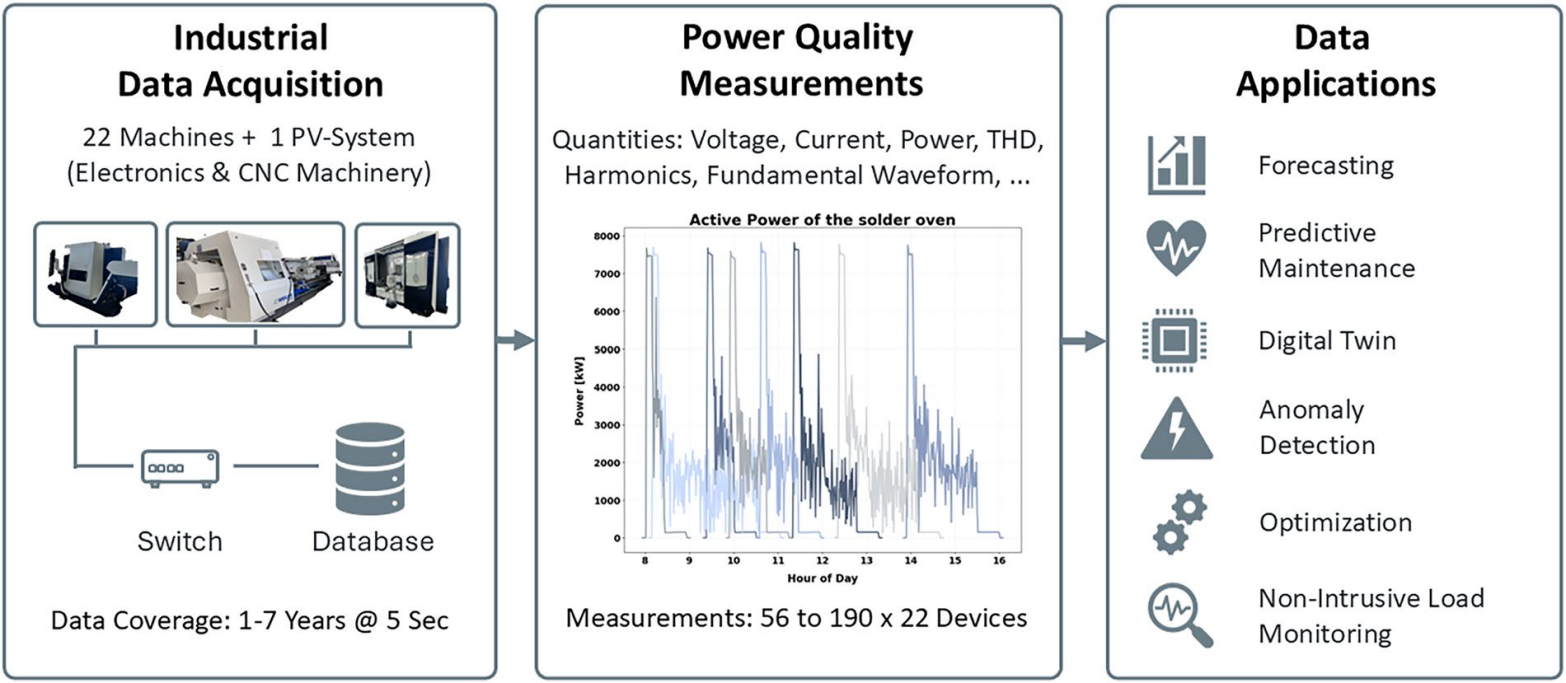
Schematic representation of the data acquisition and processing workflow.

Building on the methodological foundation of the HIPE dataset, we present a new and substantially extended dataset^22^ comprising high-resolution measurements from 22 industrial machines and a grid-connected PV system, collected continuously from 2018 to 2024 (see Fig. 1). The recordings cover a broad spectrum of industrial processes, operating conditions, and electrical configurations across two distinct facilities, enabling detailed comparative analyses. Each device provides up to 190 synchronized electrical quantities, including voltages, currents, active and reactive power, harmonic spectra, and total harmonic distortion, alongside rich contextual metadata on machine type, electricity prices, grid emissions, and weather conditions. This dataset is intended to serve as a comprehensive long-term benchmark for industrial energy research. It facilitates studies on carbon- and price-aware optimization of industrial processes, anomaly detection, and advanced methods in NILM, digital twins, and forecasting, which are currently constrained by short observation periods and limited measurement diversity.

## Methods

In this section, we describe our experimental setup, data acquisition process, monitored machines, and recorded measurements. Further, we detail the postprocessing steps applied to ensure data quality and reproducibility.

### Experimental Setup

The measurements were conducted in two industrial research facilities located at Campus North of the Karlsruhe Institute of Technology (KIT) in Eggenstein-Leopoldshafen, Germany (49.091741^°^ N, 8.428385^°^ E). Together, these sites provide complementary industrial environments with distinct operational characteristics and electrical behaviors.

The Electronic packaging and interconnects (EPI) facility focuses on research and small-batch manufacturing in electronics and power electronics. Its equipment includes assembly lines for printed circuit boards, soldering and bonding stations, and characterization benches for power modules. A rooftop PV system allows for the study of local generation-consumption interactions under realistic operating conditions. The Technic House (TEC) facility provides precision manufacturing and prototyping services for scientific instrumentation. It operates modern five-axis Computerized Numerical Control (CNC) machining centers, lathes, milling machines, and eroding systems. The workshop supports highly individualized fabrication tasks, resulting in irregular and diverse electrical load profiles.

Measurements at EPI are recorded continuously from 1 January 2018 to 31 December 2024, while TEC is monitored from 1 January 2024 to 31 December 2024. The shorter recording period at TEC reflects later instrumentation and the associated commissioning process, which included site-access limitations and harmonization of measurement configurations. Each device is sampled at a 5-second resolution, and all timestamps follow the Europe/Berlin time zone (CET/CEST) to ensure temporal consistency across the dataset. Not all machines measure the same quantities due to differences in phase configuration and measurement focus: EPI emphasizes higher-order harmonics, whereas TEC records more detailed features of fundamental waveform characteristics.

### Data Acquisition

The measurement infrastructure is designed for synchronized, real-time monitoring of industrial machines and local PV generation. Each machine is connected via single- or three-phase power lines equipped with current transformers on each active conductor to enable per-phase current measurement.

Electrical metering at EPI is performed using Phoenix Contact EEM-MA600 devices, while TEC employs EEM-MA770 meters. Both are industrial-grade smart meters that sample voltage and current at 12.8 kHz for 50 Hz systems and compute true root mean square (RMS) values over consecutive 0.5-second intervals. The meters determine active and reactive power, per-phase voltages and currents, harmonic spectra up to the 63rd order, and THD with an accuracy between 0.2% and 1%. Every 5 seconds, the most recent measurements are retrieved and stored in the database.

Data transmission is performed via Modbus/TCP over a managed Ethernet network. Netgear GS305E switches ensure stable, low-latency communication through VLAN segmentation and Quality-of-Service prioritization. All meters are connected via Cat 6 cabling to a central router that forwards the data to a local SQL database server. Real-time communication with auxiliary devices, such as PV inverters, is handled through an MQTT broker using a lightweight publish-subscribe protocol.

The database stores all measurements together with contextual metadata, including machine type and location, and is continuously mirrored and backed up to ensure data integrity. Automated threshold and missing-data alerts, together with regular monitoring and system checkups, safeguard continuous operation and data reliability. The architecture achieves full synchronization across all machines and the PV system, supporting efficient querying, visualization, and analysis through tools such as Grafana.

### Monitored Machines

**Fig. 2:**
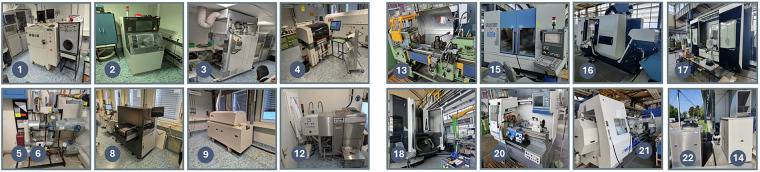
Overview of monitored machines, with numbers denoting referenced types.

To contextualize the dataset and illustrate the diversity of electrical behavior across different industrial processes, we provide a concise overview of all monitored machines. Table 2 lists all machines, specifying their name, manufacturer and model, physical location within the EPI or TEC facility, and the corresponding dataset identifier. Fig. 2 illustrates selected machines in their production environment. We further briefly describe each machine’s function, typical operation, and characteristic load behavior, with detailed technical specifications available at the respective manufacturers.

**Table 2:** Overview of all monitored devices, including machine type, manufacturer, facility location, and corresponding dataset identifier.

Nr.	Machine	Manufacturer and Type	Location	Dataset ID
1.	Chip press	HML MP 50 1 VK	EPI	EPI_ChipPress
2.	Chip saw	DISCO DAD 3240	EPI	EPI_ChipSaw
3.	High temperature oven	ATV PE0 604	EPI	EPI_HighTempOven
4.	Pick and place machine	ESSEMTEC Paraquda PARA-4	EPI	EPI_PickAndPlace
5.	Pump station 1	—	EPI	EPI_PumpStation1
6.	Pump station 2	—	EPI	EPI_PumpStation2
7.	PV system	Sunny Tripower 8.0 - 10.0	EPI	EPI_PV
8.	Screen printer	EKRA XH STS	EPI	EPI_ScreenPrinter
9.	Solder oven	SMT XXS N_2_	EPI	EPI_SolderOven
10.	Total load (complete building)	—	EPI	EPI_TotalLoad
11.	Vacuum soldering	ATV SRO 700	EPI	EPI_VacuumSoldering
12.	Washing machine	Miele IR 6002	EPI	EPI_WashingMachine
13.	Lathe 48S	VDF Boehringer 48 S	TEC	TEC_48S
14.	Water chiller	Air Blue (HOSBV) ALFA/ CF ST 161	TEC	TEC_CFST161
15.	5-axis milling machine	Chiron 800	TEC	TEC_Chiron800
16.	Lathe milling machine CTX	DMG Mori CTX Beta 800TC	TEC	TEC_CTX800TC
17.	5-axis milling machine DMF	DMG Mori DMF 300/8	TEC	TEC_DMF3008
18.	5-axis milling machine DMU	DMG Mori DMU 125 monoBLOCK	TEC	TEC_DMU125MB
19.	Milling machine DNG	Deckel / Maho DNG 50 evo	TEC	TEC_DNG50evo
20.	Lathe E30D2	Weiler E 30 D2	TEC	TEC_E30D2
21.	Lathe E110	Weiler E110 X 4500	TEC	TEC_E110
22.	Liquid cooler	KTK JWA 24 S/IK/P/A	TEC	TEC_JWA24
23.	Wire EDM machine	Mitsubishi Electric MV 2400 R	TEC	TEC_MV2400R

1. **Chip Press (EPI)**^23^: The HML MP 50 1 VK chip press combines electrically heated zones with hydraulic pressure up to 150 tons for multilayer packaging. Power demand peaks during heating and pressing phases, while harmonics arise from drive control and thermal regulation circuits.
2. **Chip Saw (EPI)**^24^: The DISCO DAD 3240 is a single-axis precision dicing saw, cutting a wide range of materials using a high-speed spindle. Energy consumption varies with spindle load and feed rate, showing harmonic distortion from inverter-based drives.
3. **High Temperature Oven (EPI)**^25^: The ATV PEO 604 furnace operates up to 1000 ^°^C. Its profile exhibits ramp-up peaks and stable holding phases, with harmonics from heater controllers, auxiliary blowers, and pumps.
4. **Pick and Place Machine (EPI)**^26^: The ESSEMTEC Paraquda PARA-4 is an automated assembly system for high-speed placement. The load alternates between idle and active placement cycles, producing cyclic patterns with harmonic content from motor drives.
5. **Pump Station 1 (EPI)**: Infrastructure subsystem for process liquid transport. Power demand depends on flow rate and pressure setpoints, producing intermittent load cycles during activation.
6. **Pump Station 2 (EPI)**: Secondary pumping infrastructure with comparable operating principles to Pump 1, serving separate circuits or redundancy functions. Consumption follows process scheduling and flow dynamics.
7. **PV System (EPI)**^27^: The 10.72 kWp rooftop PV installation employs SMA Sunny Boy 3.0 1AV-41 and Sunny Tripower 8.0 inverters for grid feed-in. Generation follows solar irradiance with a smooth diurnal profile.
8. **Screen Printer (EPI)**^28^: The EKRA XH STS stencil printer applies solder paste. Power consumption scales with print frequency, with harmonics introduced by motion control drives.
9. **Solder Oven (EPI)**^29^: The SMT XXS N_2_ reflow oven performs multi-zone soldering under nitrogen atmosphere. Load profiles are dominated by heating and convection units, with harmonics from fan drives.
10. **Total Load (EPI)**: Central measurement of the building’s aggregated power demand, including production and auxiliary systems. The profile represents the superposition of diverse machine loads and harmonic sources.
11. **Vacuum Soldering System (EPI)**^30^: The ATV SRO 700 uses infrared heating and vacuum processing for soldering up to 450 ^°^C. Power peaks occur during lamp heating and pump cycles, with transients from switching events.
12. **Washing Machine (EPI)**^31^: The Miele IR 6002 industrial washer integrates circulation pumps, heating, and drying units. Energy demand follows wash-cycle phases, while harmonics stem from inverter-controlled pumps and fans.
13. **Lathe 48 S (TEC)**^32^: The VDF Boehringer 48 S lathe performs precision turning with a servo-driven spindle. Energy use depends on material load and feed rate, with harmonics from variable-speed motor drives.
14. **Water Chiller CF ST 161 (TEC)**^33^: The Air Blue ALFA/CF ST 161 chiller regulates coolant temperature for machining processes. Power demand cycles with compressor operation, exhibiting harmonic distortion from drive systems.
15. **5-Axis Milling Machine Chiron 800 (TEC)**^34^: The Chiron 800 CNC mill enables complex multi-axis machining. Load variations follow spindle torque and tool complexity, with harmonics from coordinated servo drives.
16. **Lathe Milling Machine CTX 800 TC (TEC)**^35^: The DMG Mori CTX Beta 800 TC combines turning and milling in a single setup. Power profiles depend on tool engagement, showing harmonics from simultaneous drive operation.
17. **5-Axis Milling Machine DMF 300/8 (TEC)**^36^: The DMG Mori DMF 300/8 machining center handles large components with continuous 5-axis control. Energy demand correlates with cutting load and feed rate, producing pronounced transients from high-power drives.
18. **5-Axis Milling Machine DMU 125 MB (TEC)**^37^: The DMG Mori DMU 125 monoBLOCK provides universal 5-axis machining. Electrical load is driven by spindle torque and synchronized axis motion, with harmonic content from coordinated drive systems.
19. **Milling Machine DNG 50 evo (TEC)**^38^: The Deckel Maho DNG 50 evo CNC mill performs precision cutting with servo-driven axes and a variable-speed spindle. Energy use reflects acceleration and tool load, while harmonics originate from motor inverters.
20. **Lathe E 30 D2 (TEC)**^39^: The Weiler E 30 D2 precision lathe processes medium to large components using high-torque spindle drives. Load variations correspond to cutting depth and spindle acceleration, producing harmonics from variable-frequency drives.
21. **Lathe E 110 X 4500 (TEC)**^39^: The Weiler E 110 heavy-duty lathe performs longitudinal turning of large parts. Energy consumption scales with feed rate and workpiece size, with harmonics from drive control systems.
22. **Liquid Cooler JWA 24 S (TEC)**^40^: The KTK JWA 24 S/IK/P/A liquid cooling unit stabilizes thermal conditions during machining and EDM operations. Power cycles with compressor activity, exhibiting harmonic distortion from compressor and pump drives.
23. **Wire EDM Machine MV 2400 R (TEC)**^41^: The Mitsubishi MV 2400 R wire electrical discharge machine erodes conductive materials using pulsed discharges. Energy use follows spark frequency and wire feed, producing pulsed signatures and harmonics.

### Recorded Measurements

The dataset contains 257 distinct electrical quantities, though individual machines record different subsets depending on their phase configuration and metering focus. Table 3 summarizes the electrical setup of all devices, including phase count, nominal voltage, rated current, observation period, recorded channels, and total valid data points. For completeness, missing rated currents are estimated from the measured current values. TEC machines consistently record 125 quantities, while three-phase and single-phase devices at EPI capture 190 and 56 quantities, respectively. The main distinction lies in harmonic resolution, as EPI meters measure up to the 31st harmonic, whereas TEC meters capture only up to the 5th but provide enhanced fundamental-wave descriptors.

**Table 3:** Electrical configuration and measurement scope for all monitored machines; missing values are estimated from data.

Nr.	Machine	Phases	Supply Voltage	Rated Current	Years	Channels	Data Points
1.	Chip press	3P	400 V	50 A	7	190	6.94 billion
2.	Chip saw	3P	400 V	6 A	7	190	7.42 billion
3.	High temperature oven	3P	400 V	32 A (estimate)	7	190	7.22 billion
4.	Pick and place machine	1P	230 V	5 A	7	55	2.61 billion
5.	Pump station 1	3P	400 V	34 A (estimate)	7	190	6.03 billion
6.	Pump station 2	3P	400 V	36 A (estimate)	7	190	6.14 billion
7.	PV system	3P	400 V	16 A (estimate)	1	2	2 million
8.	Screen printer	1P	230 V	5.2 A	7	55	2.38 billion
9.	Solder oven	3P	400V	29 A	7	190	7.45 billion
10.	Total load	3P	400 V	160 A (estimate)	7	190	6.62 billion
11.	Vacuum soldering	3P	400 V	14 A	7	190	6.65 billion
12.	Washing machine	3P	400 V	18 A (estimate)	7	190	6.55 billion
13.	Lathe 48S	3P	400 V	40 A (estimate)	1	125	727 million
14.	Water Chiller	3P	400 V	40 A (estimate)	1	125	732 million
15.	5-axis milling machine	3P	400 V	90 A (estimate)	1	125	732 million
16.	Lathe milling machine CTX	3P	400 V	100 A	1	125	732 million
17.	5-axis milling machine DMF	3P	400 V	80 A	1	125	730 million
18.	5-axis milling machine DMU	3P	400 V	125 A	1	125	727 million
19.	Milling machine DNG	3P	400 V	80 A	1	125	732 million
20.	Lathe E30D2	3P	400 V	15 A	1	125	726 million
21.	Lathe E110	3P	400 V	80 A	1	125	726 million
22.	Liquid cooler	3P	400 V	45 A (estimate)	1	125	725 million
23.	Wire EDM machine	3P	400 V	70 A	1	125	717 million

**Fig. 3:**
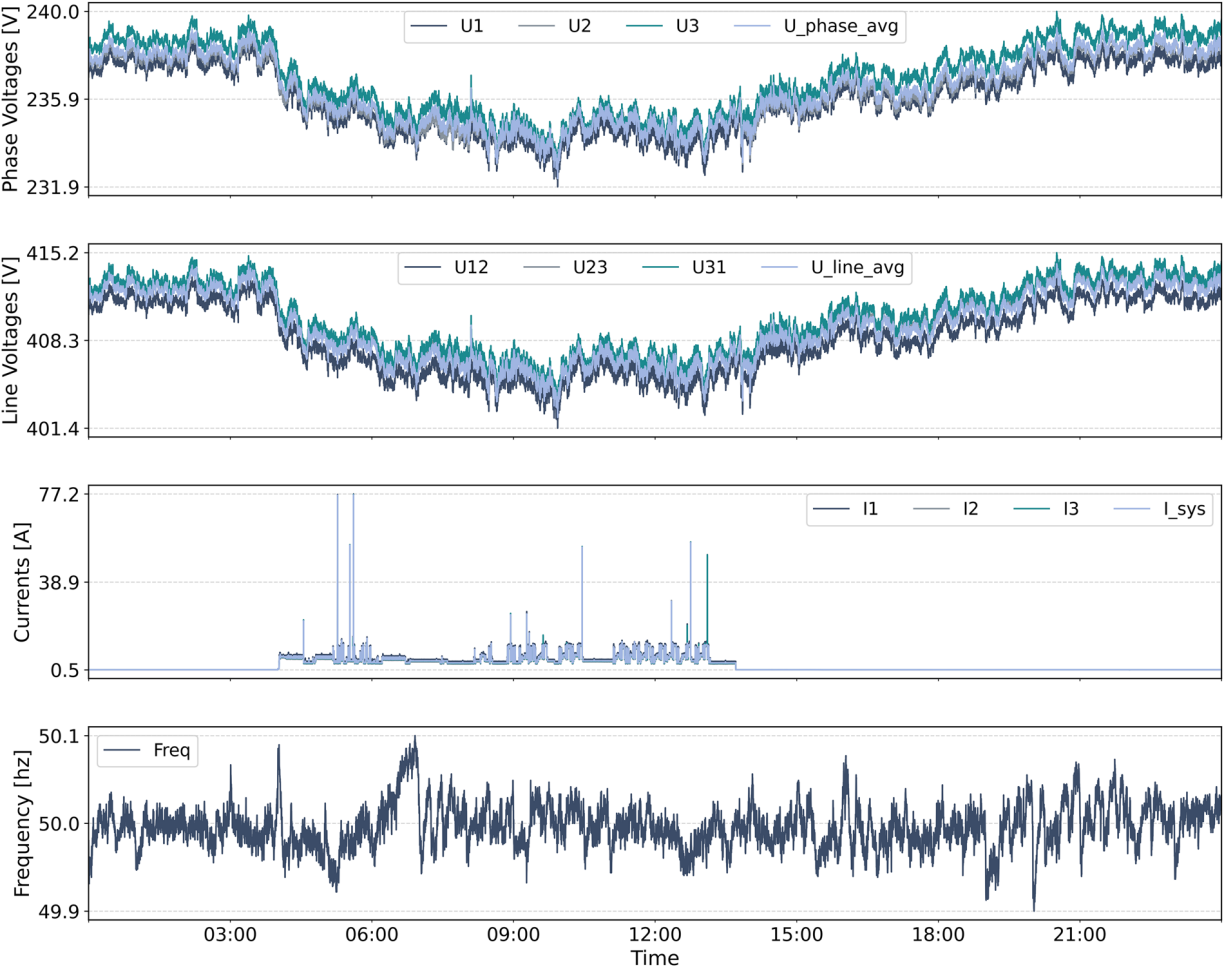
Power measurements of TEC_Chiron800 collected on 2024-05-15 with 5 s sampling.

**Fig. 4:**
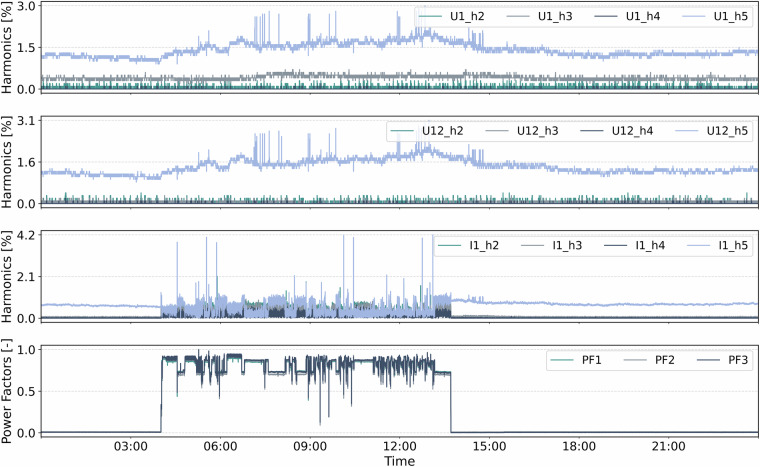
Power quality measurements of TEC_Chiron800 collected on 2024-05-15 with 5 s sampling.

The following section groups and describes the measured quantities. The highlighted identifiers follow the naming convention used consistently across the dataset. Figs. 3 and 4 illustrate one exemplary day of selected energy profiles for the machine TEC_Chiron800 and help contextualize the measurements.

- **Voltages [V]**: The dataset reports the RMS values of the phase-to-neutral voltages (**U1,** **U2,** **U3**) and the line-to-line voltages (**U12,** **U23,** **U31**). Additionally, we include **U_phase_avg** representing the mean of the three phase-to-neutral voltages and **U_line_avg** indicating the mean of the three line-to-line voltages. These quantities provide a compact characterization of system-level voltage levels and are crucial for voltage stability analysis.
- **Currents [A]**: The RMS currents in the three phase conductors (**I1,** **I2,** **I3**) and in the neutral conductor (**IN**) are recorded. The system current **I_sys** denotes the mean of the three-phase currents. These measurements provide insight into the load distribution across phases and potential neutral imbalances in the system.
- **Voltage Angles** [^°^]: The absolute angles of the voltage phasors **(Angle**_**U1,** **Angle**_**U2,** **Angle**_**U3)** represent the phase position of each line voltage with respect to a time reference. These values characterize the phase sequence and potential imbalances in the supply.
- **Rotating field [-]**: The rotating field direction (**RotField**) indicates the sequence of the three-phase system: +1 for clockwise, -1 for counterclockwise, and 0 for undefined.
- **Power Quantities - Active, Reactive, Apparent [W, var, VA]**: Active power (**P1,** **P2,** **P3**) quantifies the real energy transfer per unit time in each phase (their sum is denoted as **P_total**). Reactive power is captured both per phase (**Q1,** **Q2,** **Q3**) and as the sum **Q_total**. Apparent power is likewise given per phase (**S1,** **S2,** **S3**) and in total as **S_total**.
- **Power Factor [-]**: The power factor is defined as the ratio of active to apparent power. It is reported per phase (**PF1,** **PF2,** **PF3**) and as system-level values (**PF_total**). Power factors close to 1 indicate efficient energy usage, while lower values imply increased reactive power. Sign conventions indicate inductive (>0) or capacitive (<0) characteristics.
- **Load Type Indicators [-]**: The load type indicators (**LoadType1,** **LoadType2,** **LoadType3**) are discrete values encoding the predominant reactive behavior in each phase: +1 indicates inductive, -1 capacitive, and 0 purely resistive loads.
- **Frequency [Hz]**: The network frequency (**Freq**) denotes the instantaneous frequency of the voltage waveform, typically expected to remain close to the grid frequency of 50 Hz.
- **Operating Time [h]**: The total operating duration is recorded as **OpHours**, measured as the cumulative time during which the monitoring system has been active.
- **Total Harmonic Distortion (THD) [%]**: THD values are provided for line voltages (**THD_U12,** **THD_U23,** **THD_U31**), phase voltages (**THD_U1,** **THD_U2,** **THD_U3**), and currents (**THD_I1,** **THD_I2,** **THD_I3,** **THD_IN**). THD is defined as the RMS sum of all harmonic components normalized to the fundamental component. It quantifies waveform distortion and is a key indicator of power quality.
- **Harmonics of Voltages and Currents**: For each phase and line voltage (U1, U2, U3, U12, U23, U31) and each current (I1, I2, I3, IN), the dataset includes relative magnitudes of the 2nd to 31st harmonics (e.g., **U1_h2** to **U1_h31**). This detailed spectral information enables a precise harmonic decomposition and supports diagnostics of non-linear loads.
- **Fundamental Wave Quantities**: All major quantities, voltages, currents, powers, power factors, angles, and load types, are also calculated based solely on the fundamental frequency component. These are denoted with a suffix _f (e.g., **U1_f,** **P1_f,** **PF_total_f**). They offer a cleaner view of the ideal sinusoidal response and isolate the contribution of harmonic distortion.
- **PV Quantities**: For the PV system we further report **AC_ActivePower** [W] and **DailyYield** [Wh].

### Data Postprocessing

To facilitate the data usage, we structure, compress, and align the files to produce a coherent dataset. The procedures below are designed to improve usability across common workflows (forecasting, anomaly detection, power-quality analysis).

- **Structuring**. We partition the dataset into compact files to enable efficient access on standard hardware. Individual uncompressed files range from 100 to 200 MB, enabling processing even on systems with limited memory. After evaluating alternative layouts (e.g., grouping by signal family or by shorter time spans), we adopt the hierarchy: machine  → measurement  → year. Thereby, each file corresponds to one calendar year of measurements for a specific machine and measurement.
- **Compression**. All files are stored in the .csv.xz format to minimize storage requirements and improve data transfer efficiency. The xz compression was selected after evaluating several alternatives (gzip, bz2, zip, zstd) due to its superior compression ratio, achieving about a tenfold reduction in file size, and its broad compatibility with computing tools such as the Python pandas library.
- **Naming and format**. Filenames, directories, and column headers follow a unified scheme across EPI and TEC. Timestamps are provided in a column named *WsDateTime* (CET/CEST). Measurement columns have the name of the measurement (e.g., U12, I3). Numerical values are stored in 32-bit floating-point format (float32), which reflects the sensor precision.
- **5 second alignment**. All time series are reindexed to a uniform 5-second grid per calendar year. For each record, we select the nearest grid time if the absolute difference is  < 5 seconds. If two records map to the same grid point, we retain the closest. We verify that alignment preserves distributional properties by comparing mean, standard deviation, and the 1st/99th percentiles before and after alignment. The unified time stamps facilitate machine comparisons and measurement validation. Note that we also provide the unprocessed dataset^42^ in case no alignment is wanted.
- **Exogenous features**. We provide separate files from external sources (all with CC BY 4.0 license) for electricity prices, grid emissions, weather variables, and holidays for use cases like energy forecasting, cost optimizations, or emission reductions.
- **Metadata**. The dataset includes detailed metadata with descriptions of all machines, measurement variables, exogenous features, and missing files. It also contains summaries of data availability showing the share of valid entries, and analyses of data gaps describing their duration and position within each measurement run.

## Data Records

The postprocessed dataset is available under a CC BY 4.0 license on RADAR4KIT^22^. To ensure transparency, the original unprocessed dataset is also provided^42^. Access to both versions allows users to reproduce the full postprocessing workflow and test algorithm robustness to data issues such as time-step misalignment or outliers. However, we highly recommend the cleaned dataset for empirical analyses.

The cleaned dataset comprises 44,865 files organized into 26 top-level directories to support both complete and partial downloads. Users can download the full repository or select specific folders via the repository content panel, which is particularly useful when local storage is limited. These directories include 22 machine folders, one PV system folder, one validation folder, and two auxiliary folders containing metadata and exogenous inputs. The 22 machine folders and the PV system folder contain 14,155 cleaned measurement files. The validation folder dataset_clean_validation mirrors the machine and PV folder structure and provides two companion files for each measurement: a statistics file (_stats.csv) summarizing basic metrics such as count, mean, standard deviation, minimum, maximum, and selected quantiles, and a missing-data file (_missing.csv) listing data gap times, durations, and coverage. These companion files facilitate rapid screening and aggregation, whereas detailed analyses should rely on the cleaned measurement files.

All measurement-containing folders follow the same three-level hierarchy: machine → measurement → year. Machine folders use the identifiers listed in Table 2 (e.g., EPI_PickAndPlace). Each measurement forms a subfolder (e.g., U12) containing up to seven compressed CSV files, one per year (e.g., 2018_U12.csv.xz). For instance, the U12 measurement from machine EPI_ChipPress for the year 2018 and its validation files are located at:

$$\begin{array}{c}{\mathtt{EPI}}\_{\mathtt{ChipPress/U12/2018}}\_{\mathtt{U12.csv.xz}}\\ {\mathtt{dataset}}\_{\mathtt{clean}}\_{\mathtt{validation/EPI}}\_{\mathtt{ChipPress/U12/2018}}\_{\mathtt{U12}}\_{\mathtt{stats.csv}}\\ {\mathtt{dataset}}\_{\mathtt{clean}}\_{\mathtt{validation/EPI}}\_{\mathtt{ChipPress/U12/2018}}\_{\mathtt{U12}}\_{\mathtt{missing.csv}}\end{array}$$

**Fig. 5:**
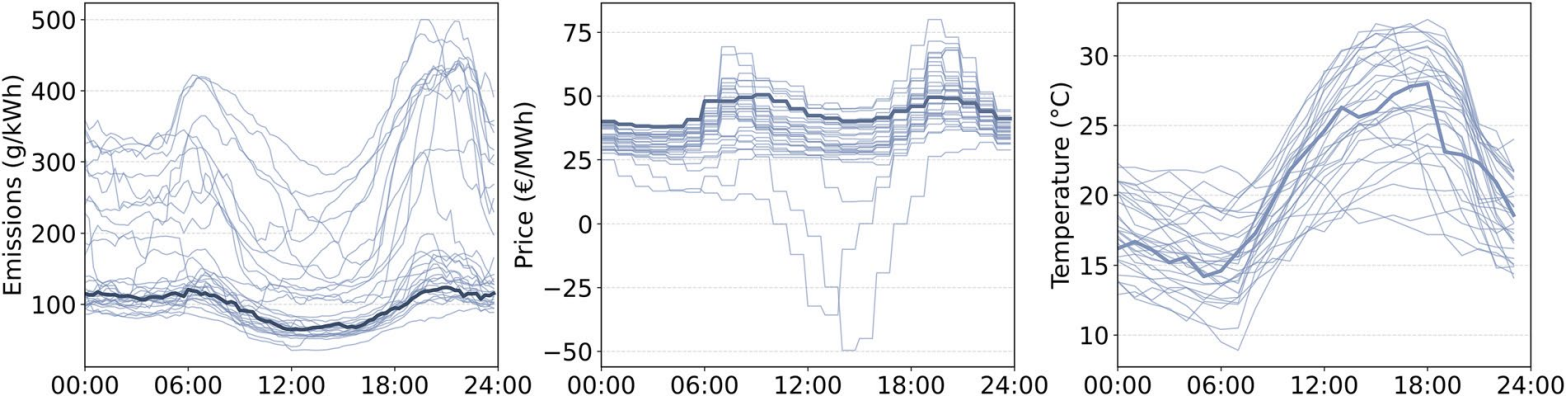
Daily profiles for emissions, electricity price, and air temperature for August 2019: thin blue lines show each day, while the bold line highlights the 2019-08-02 profile.

Beyond the measurement and validation directories, the dataset provides two auxiliary folders, 00meta_data and 01exogenous_data, all under CC BY 4.0 license. The folder 01exogenous_data contains the following external data sources that complement the machine-level measurements and are essential for modeling, forecasting, and interpretation. Figure 5 illustrates selected exogenous features.

- electricity_emissions_15min_transnetbw_2018_2024.csv: Contains 15-minute electricity generation data by technology (MW) and corresponding emission intensities (g CO_2_/kWh) for the TransnetBW control area. These values enable analyses linking industrial consumption with grid carbon intensity and generation structure. We obtain the electricity generation time series from the SMARD platform of the German Federal Network Agency under CC BY 4.0 license. To reproduce the time series, open SMARD  → Downloadcenter  → Download market data^43^ and select Electricity generation as the main category, Actual generation as the data category, TransnetBW as the control area, Quarter-hour as the temporal resolution, and export the required study period as CSV. Technology-specific emission factors were taken from the German Federal Environment Agency^44^ and merged with the SMARD generation records by technology and timestamp.
- electricity_prices_15min_de_2018_2024.csv: Provides Germany-wide day-ahead wholesale electricity prices (EUR/MWh) with 15-minute resolution. These data support applications such as cost optimization, demand response evaluation, and price-based flexibility modeling. We obtain the electricity price time series from SMARD under CC BY 4.0 license. To reproduce the time series, open SMARD  → Downloadcenter  → Download market data^43^ and select Market as the main category, Day-ahead prices as the data category, TransnetBW as the control area, Quarter-hour as the temporal resolution, and export the required study period as CSV.
- holidays_karlsruhe_bw_2018_2024.csv: Lists official public holidays in Karlsruhe (Baden-Württemberg), including the date, weekday, a holiday indicator, and the holiday name. The series can be reproduced programmatically using the Python package *holidays* (MIT license)^45^ by selecting the country code DE (Germany) and the subdivision BW (Baden-Württemberg) for the desired timeframe. The resulting derived table is included in our dataset under the CC BY 4.0 license.
- weather_data_karlsruhe_hourly_2018_2024.csv: Provides hourly meteorological observations from the German Weather Service, including air temperature, humidity, wind speed and direction, air pressure, global radiation, sunshine duration, cloud cover, visibility, and precipitation^46^. These variables support analyses of weather-related effects on energy consumption and PV generation. The data are sourced from the DWD Climate Data Center Open Data repository under CC BY 4.0 and were retrieved programmatically using the Python library *wetterdienst*^46^. We queried hourly observations for Karlsruhe (49.0069^°^N, 8.4037^°^E) during the study period. For each variable, we first used the station closest to Karlsruhe. If coverage was insufficient, we searched for stations within 80 km using the filter_by_distance function and selected the station with the highest proportion of non-missing hourly values.
- weather_data_stations_used.csv: Provides location metadata for the time series in weather_data_karlsruhe_hourly_2018_2024.csv by documenting the DWD station selected for each weather variable during data extraction. The file reports the station ID, station name, federal state, distance to Karlsruhe, elevation, and geographic coordinates^46^. This metadata enables transparent traceability of the data source and supports spatial comparability of the observations.

The 00meta_data folder provides structured descriptions of machines, measurements, external data sources, and data quality assessments, thereby supporting reproducible analyses and automated data handling. Here, we include:

- 00measurement_details.json: Provides a complete catalog of all measurements, including physical units and human-readable descriptions, serving as a reference for variable interpretation.
- 00machine_details.json: Describes each monitored machine with manufacturer information, measurement configuration, phase setup, observation period, total number of recorded timesteps, and the share of valid data.
- 00exogenous_details.json: Documents all exogenous data files, including content description, temporal resolution, time coverage, column names, and units, ensuring consistent integration with machine-level measurements.
- 01removed_files_registry.csv: Lists all files excluded during data curation together with the associated machine, measurement, year, reason for removal, and original file path, enabling full traceability of preprocessing decisions.
- 02data_availability.csv: Summarizes the fraction of valid data points per file relative to the 5-second reference grid, providing a quick overview of data completeness.
- 03gap_composition_by_machine.csv: Characterizes missing data for each machine, reporting total gap duration, distribution across duration ranges (short interruptions to multi-week outages), and the position of gaps within the time series (beginning, middle, or end).

The dataset does not include direct measures of manufacturing activity or throughput, such as the number of processed boards or modules per hour, because these quantities were not recorded by the metering infrastructure and cannot be reconstructed retrospectively. As a proxy, operational intensity can be inferred from the electrical time series using pattern-based methods, for example, change-point detection to separate idle and active operating regimes.

## Technical Validation

We systematically validate our data using the following methods to ensure structural integrity, temporal consistency, and statistical plausibility across all machines and years.

**Fig. 6:**
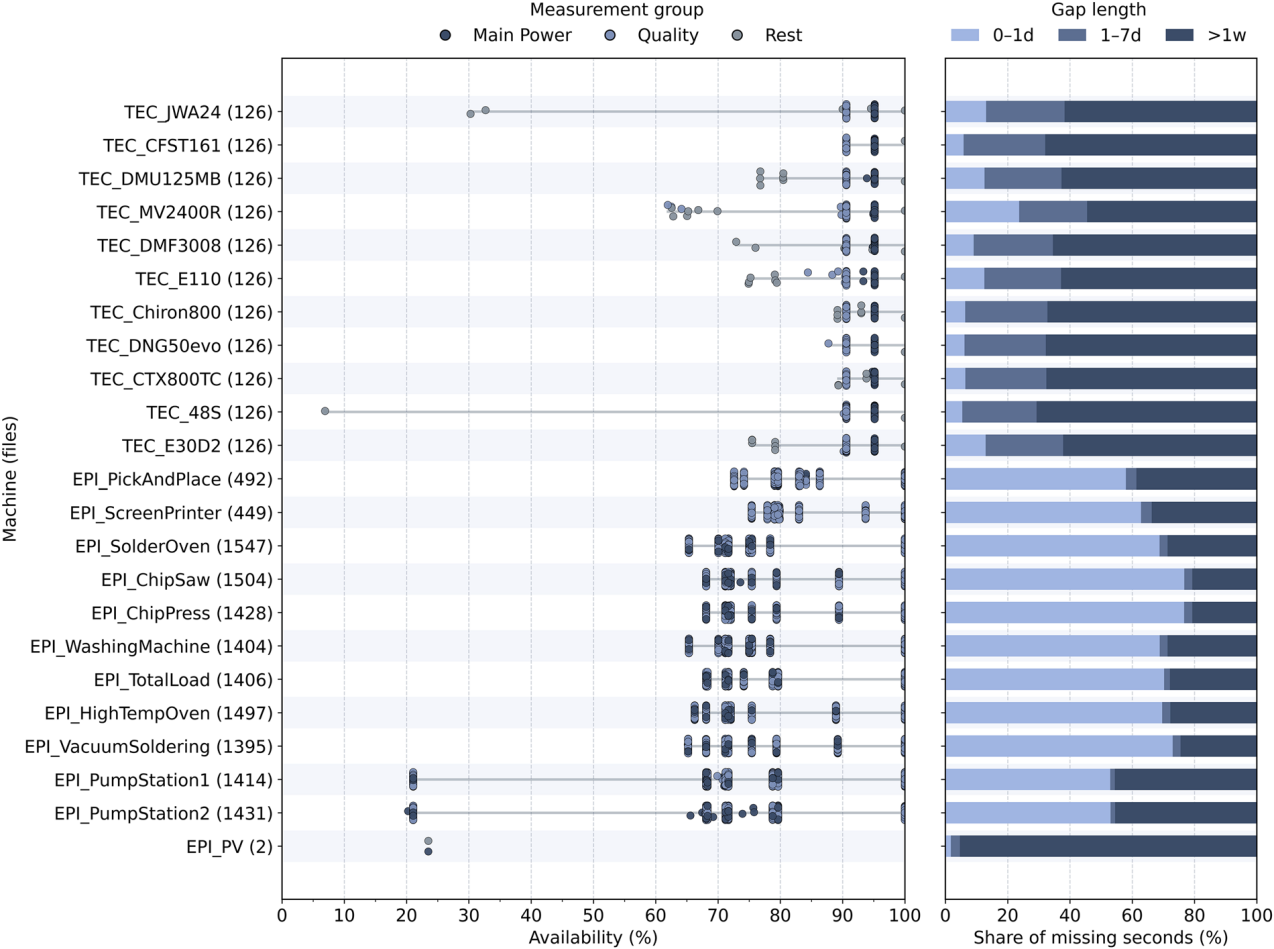
Availability of power and quality measurements across all monitored machines (left) and distribution of missing data durations (right). Each dot represents one measurement file.
- **Structurally empty measurements**. Certain higher-order harmonic channels are included in the metering configuration but record no measurable activity throughout an entire year. A file is classified as structurally empty if all statistical indicators (minimum, maximum, mean, and standard deviation) equal zero. Applying this criterion identifies 1,352 files. For transparency, all affected entries are documented in 01removed_files_registry.csv, and their locations are replaced by explicit _EMPTY placeholders containing only the standard header. This approach preserves the directory structure for automated access while minimizing storage and processing requirements.
- **Validating physical limits**. Each signal is validated to ensure its statistical properties fall within physically plausible ranges. Values outside defined limits are replaced with NaN. Validation is applied consistently across dataset_clean using a combination of global and machine-specific thresholds derived from rated current, nominal voltage, and device characteristics. Phase and neutral currents are limited to slightly above the rated current, while apparent and active power values are restricted according to machine capacity. Acceptable voltage ranges are 200.9-260.0 V for phase-to-neutral and 350.0-450.0 V for line-to-line measurements. The grid frequency is expected between 49 Hz and 51 Hz, phase angles between  −180^°^ to 180^°^, and dimensionless indicators such as power factor and load type between -1 and 1. Harmonic magnitudes and total harmonic distortion are constrained to 0-100%. Two machines at EPI show persistent outliers, long data gaps, and implausible values across multiple channels. We therefore excluded both machines (Vacuum Pump 1 (Rietschle VLT 15) and Vacuum Pump 2 (Leybold Ecodry 35 Plus)) from this validated dataset. However, they remain available in the raw dataset^42^.
- **Validation of physical relationships**. In addition to range checks, all signals were verified for internal consistency based on fundamental electrical relationships. Each equation was evaluated using mean values of total and fundamental quantities within a tolerance of 20 %. This tolerance is chosen conservatively because the checks are automated on file-level aggregates, which can vary due to missing-data patterns with different lengths and timings. The objective is to identify gross inconsistencies without discarding plausible files, while enabling stricter file-wise filtering in a subsequent step if necessary. Fundamental consistency was first checked to ensure that total quantities exceed or equal their corresponding fundamental components (*I* ≥ *I*_*f*_, *U* ≥ *U*_*f*_, ∣*P*∣ ≥ ∣*P*_*f*_∣). Aggregation consistency was then verified through phase-sum relations (*P*_total_ ≈ *P*_1_ + *P*_2_ + *P*_3_, *Q*_total_ ≈ *Q*_1_ + *Q*_2_ + *Q*_3_, *S*_total_ ≈ *S*_1_ + *S*_2_ + *S*_3_) and average voltage relations (*U*_phase,avg_ ≈ (*U*_1_ + *U*_2_ + *U*_3_)/3, *U*_line,avg_ ≈ (*U*_12_ + *U*_23_ + *U*_31_)/3). Finally, the power triangle relations ($S=\sqrt{{P}^{2}+{Q}^{2}}$, ${S}_{f}=\sqrt{{P}_{f}^{2}+{Q}_{f}^{2}}$) were validated for both total and fundamental components.
- **Operational validation and monitoring**. To ensure data reliability, sensor calibration is verified to prevent false measurements. Thresholds for key quantities such as voltage, current, and power trigger automated alerts when deviations occur. Operator and maintenance feedback supports anomaly interpretation, while continuous monitoring enables visual inspection. For a first overview, Figure 7 presents the weekly active power profiles of all monitored machines, highlighting the diversity in operating patterns across different processes and facilities. Figure 8 further depicts the harmonic spectra of the line-to-line voltage (U12) and phase current (I1) for each device.

- **Schema and integrity checks**. The dataset structure is validated to ensure consistency and correctness across all components. All file names adhere to the convention YYYY_MEASUREMENT.csv.xz. Each file must contain two columns, WsDateTime and the corresponding measurement identifier, with standardized data types (timestamp and floating-point). Record counts are verified against the expected number for a 5-second resolution, and units and formatting are harmonized across EPI and TEC. Any detected inconsistencies are systematically identified and corrected.
- **Missing data and availability**. Missing data mainly arise from system reboots, power outages, and interruptions during software updates, whereas longer gaps typically result from planned maintenance and the subsequent reconfiguration of the data acquisition setup, including network changes. For each file, data completeness is assessed at a 5-second resolution by determining valid entry coverage, identifying gap start and end times, and quantifying gap durations. Results are aggregated per machine and measurement, with detailed reports (*_missing.csv) provided in dataset_clean_validation. No interpolation or imputation is applied, ensuring that the dataset contains only measured values. Figure 6 summarizes, for each machine, the number of available files, data availability in percent for power and power-quality quantities, and the distribution of gap durations (shorter than one day, one to seven days, and longer than one week).

**Fig. 7:**
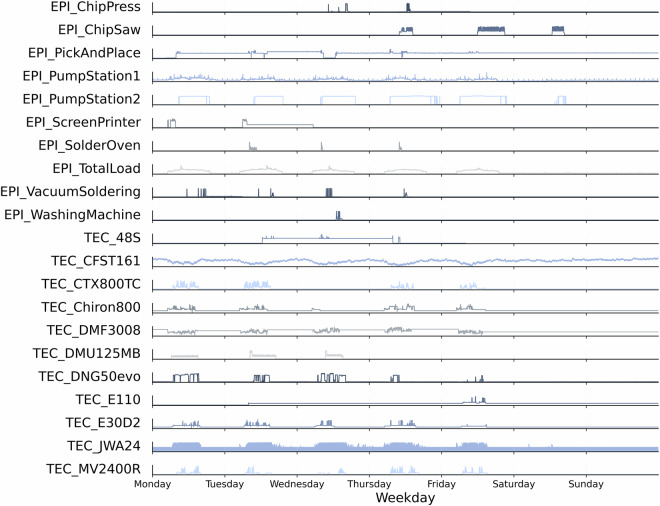
Power profiles of all machines during a representative week.

**Fig. 8:**
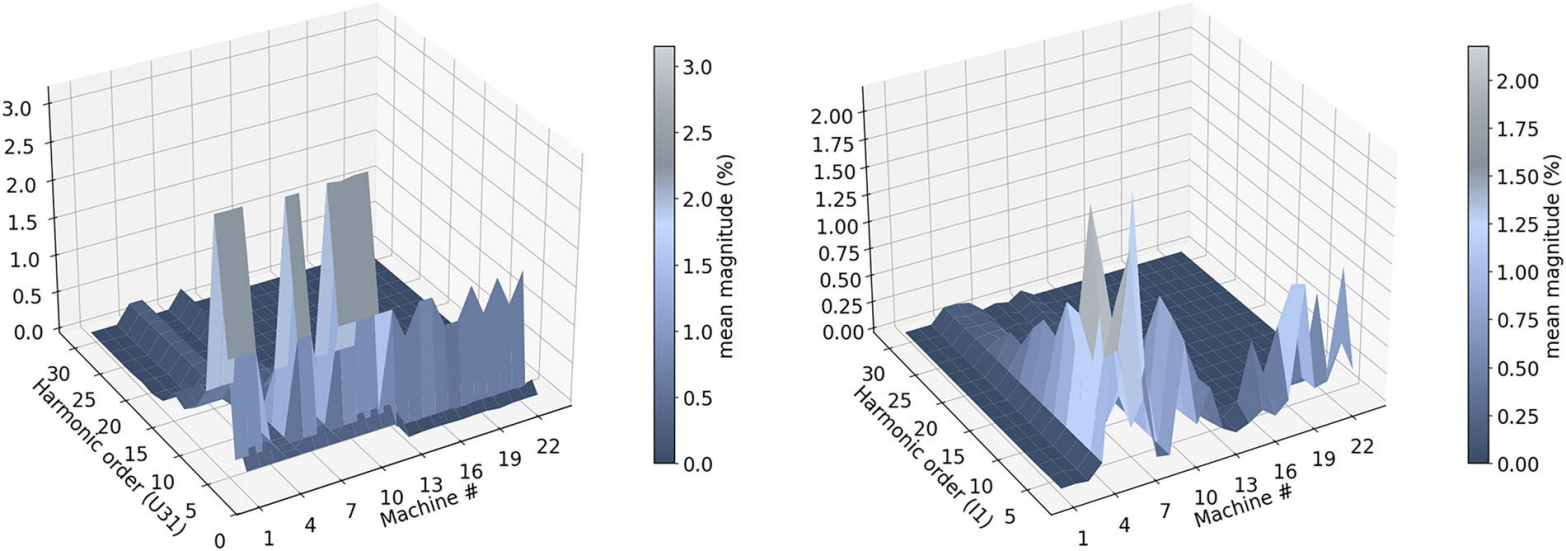
Comparative harmonic behavior of line-to-line voltage U12 and phase current I1 (orders 2-31).

## Usage Notes

We encourage the use of this dataset for a broad range of industrial energy research and analytics applications. The detailed power measurements (active, reactive, and apparent power) enable analyses of load behavior, energy efficiency, and demand-side flexibility, supporting applications such as forecasting, energy optimization, and NILM. The voltage and current measurements facilitate the study of electrical balance, phase asymmetry, and provide a basis for power quality assessment, anomaly detection, and predictive maintenance. Combined with contextual metadata such as electricity prices, grid emissions, and weather data, the dataset supports analyses of battery storage integration and on-site PV optimization. These studies can inform strategies to reduce energy costs and carbon emissions in industrial facilities. Note that we recommend using the validated and cleaned dataset^22^ for most use cases.

## Data Availability

The dataset presented in this article is publicly available on RADAR4KIT under a CC BY 4.0 license. Two versions are provided: a cleaned and validated dataset reflecting all processing steps described in this paper (10.35097/bjdg3m3rg5jv3skk), and a raw dataset containing the original unprocessed measurements (10.35097/8efkkn0g9pcng5yd). The raw dataset contains outliers, inconsistent measurement units between machines (e.g., ampere vs. milliampere), and non-harmonized variable names (e.g., U12 vs. V12). The cleaned dataset includes comprehensive metadata and detailed measurement descriptions under the same CC BY 4.0 license. In total, it contains 44,865 files organized into 26 top-level directories, enabling both full and selective downloads via the repository content panel. The main measurement data is stored in the machine folders and the PV system folder, comprising 14,155 cleaned files arranged in a consistent three-level hierarchy: machine, measurement, and year. The cleaned dataset is recommended for empirical studies, while the raw version remains available to ensure transparency and enable full reproducibility of the preprocessing workflow.

## Code availability

All preprocessing, validation, and visualization scripts are openly available on GitHub (https://github.com/JonasSievers/SPARK-High-resolution-energy-data-from-a-Sustainable-Production-Area-in-Karlsruhe)^47^. The repository includes a detailed README describing the structure and installation procedure, and a requirements.txt file listing all dependencies. The code operates on the cleaned dataset, which should be downloaded and placed in the same directory. Example scripts illustrate data access, filtering, and analysis, enabling reproducibility and easy adaptation for custom research workflows.
